# Acceptability, feasibility and equity implications of nutritional supplementation interventions for the prevention of wasting in infants and young children: A rapid qualitative evidence synthesis

**DOI:** 10.4102/phcfm.v18i1.5137

**Published:** 2026-01-15

**Authors:** Amanda S. Brand, Marianne E. Visser, Idriss I. Kallon, Susanna S. van Wyk, Anke C. Rohwer

**Affiliations:** 1 Centre for Evidence-based Health Care, Division of Epidemiology and Biostatistics, Faculty of Medicine and Health Sciences, Stellenbosch University, Cape Town, South Africa

**Keywords:** child wasting, qualitative evidence synthesis, acceptability, feasibility, equity, nutrition interventions

## Abstract

**Background:**

Child wasting remains a challenge despite global targets to eliminate malnutrition by 2030. While the global nutrition community has traditionally focused on treatment, a range of nutrition-specific interventions to prevent child wasting are available.

**Aim:**

To conduct a rapid qualitative evidence synthesis exploring factors influencing the acceptability, feasibility and equity of preventative interventions to inform a World Health Organization (WHO) guideline on child wasting. This manuscript reports on nutritional supplementation interventions, a subsection of the broader scope of the guideline.

**Method:**

We searched MEDLINE (PubMed) (database inception to 13 June 2022) for eligible studies. We coded and synthesised findings using a ‘best fit’ framework synthesis approach and assessed methodological quality of included studies. We presented fit-for-purpose evidence to complete qualitative evidence-to-decision criteria for the WHO recommendation.

**Results:**

We included 25 articles and identified 27 themes relating to acceptability, feasibility and equity for nutritional supplementation interventions. Nutritional supplementation in children was mostly acceptable, but acceptability was mixed for other recipients. Several barriers to and facilitators of nutritional supplementation across intended recipient groups were identified, with education or information frequently emerging as facilitator. Health beliefs, as well as practical challenges, are notable barriers. Evidence on equity is sparse, but sharing practices and gender roles emerged as exacerbating factors.

**Conclusion:**

Nutritional supplementation interventions are probably acceptable, and there are facilitators of implementation; however, some barriers would also need to be considered. Information regarding equity was relatively sparse.

**Contribution:**

Our findings were used in drafting the WHO guideline recommendations on child wasting.

## Background

There are an estimated 45.4 million children with wasting (weight-for-height of < –2 standard deviations [s.d.s] of the World Health Organization [WHO] growth standards) in the world, representing 6.7% of all children under 5 years of age, of which 31.8 million have moderate wasting.^1^ Wasting, formerly known as acute malnutrition, refers to a child who is too thin for their height. In 2015, the world committed to the Sustainable Development Goals (SDGs), which incorporated the World Health Assembly (WHA) targets to reduce the proportion of children suffering from wasting to less than 5% by 2025 and less than 3% by 2030.^2^ The United Nations Agencies working on the prevention of child wasting have developed a Framework for the Global Action Plan (GAP) on Child Wasting to meet these targets.^3^ In 2017, only 29 out of 193 countries were on course to achieve the WHA 2025 target.^4^ A 2017 analysis of the WHA targets for improving maternal, infant and young child (IYC) nutrition by 2025 highlighted the lack of robust studies on effective interventions to prevent wasting and a focus in the global nutrition community on the treatment rather than the prevention of wasting.^5^ To achieve the SDG wasting targets, a fundamental policy shift is needed that increases actions and investment in the prevention of malnutrition.^3^ A range of interventions are implemented in efforts to prevent wasting, including nutrition-specific and nutrition-sensitive interventions provided to infants, children and their mothers and/or caregivers, families as well as to affected communities or groups. Nutrition-specific interventions include breastfeeding promotion and support, nutrition counselling and nutrition education as well as the provision of nutritional supplementation. Nutritional supplementation comprises general food distribution to supplement diets of households and the provision of micronutrient supplementation (MNS), complementary foods and specially formulated foods.^6^ The latter include lipid-based nutrient supplements (LNSs) and fortified blended foods (FBFs) to supplement the diets of children and mothers. Various types of LNS are used, for example, ready-to-use therapeutic food (RUTF), also called large-quantity LNS (LQ-LNS), which provides around 500 kcal per day – 1500 kcal per day and is often used to treat or prevent severe wasting. Ready-to-use supplementary food (RUSF) or medium-quantity LNS (MQ-LNS) is often used to treat or prevent moderate wasting, providing about 250 kcal – 500 kcal. Along with RUTF and RUSF, there is a third category of LNS called small-quantity LNS (SQ-LNS) that provides about 110 kcal – 130 kcal, often as a fortified spread to deliver micronutrients in a lipid formulation that aids the absorption of fat-soluble vitamins and provides additional energy. Fortified blended foods are blended foods that require cooking before consumption, usually flours cooked into a porridge (e.g. a corn-soy blend [CSB]). Enhanced FBFs are those with added micronutrients and/or milk protein (e.g. Supercereal/CSB+ or Supercereal+/CSB++). The WHO, as one of the United Nations Agencies driving the GAP on Child Wasting, will be the lead agency at a global, regional and national level for the development of normative guidance and tools to support governments on the prevention and treatment of child wasting in all contexts. To this end, the WHO developed and updated a guideline on the prevention and treatment of wasting in infants and young children (birth to 5 years of age). The WHO guideline development process formulates its recommendations using the GRADE (Grading of Recommendations Assessment, Development and Evaluation) evidence-to-decision (EtD) framework comprising defined criteria, which include acceptability, feasibility and equity.^7^ Evidence-to-decision frameworks aim to ensure that all criteria relevant to a health decision are systematically and transparently considered. They offer a structured approach for decision-makers to systematically consider the best available evidence and to make judgements informed by an awareness of the advantages and disadvantages of a given health decision.^8^ In line with WHO guideline development methods, a number of systematic reviews were commissioned for the wasting guideline, informed by the guideline questions that were scoped and prioritised by the WHO Guideline Development Group (GDG).^7^ This qualitative evidence synthesis (QES) about stakeholders’ perspectives on acceptability, feasibility and equity implications of interventions for the prevention of wasting in infants and young children is one of these commissioned reviews. The aim of this QES is to synthesise key evidence around acceptability, feasibility and equity implications of interventions to prevent wasting help inform a WHO wasting guideline.

## Methods for conducting the qualitative evidence synthesis

### Aims

The overarching objective of the QES was to identify and synthesise the qualitative evidence in the form of perspectives of key stakeholders regarding the acceptability, feasibility and equity of: (1) interventions for the prevention of wasting in infants and young children and (2) the delivery of these interventions to help inform a WHO guideline. Interventions within the guideline scope were divided into four overarching categories; this article focuses on perspectives on the acceptability, feasibility and equity of nutritional supplementation intervention. We conducted a rapid QES using the ‘best fit’ framework synthesis approach and reported the findings as per the ENTREQ reporting guidelines.^9,10,11^ The protocol was prospectively registered with PROSPERO (PROSPERO 2022 CRD42022351360).

### Criteria for considering studies for this review

The research question was formulated according to the SPIDER tool for QES to assess acceptability, feasibility and equity according to the GRADE EtD framework in GRADEPro.^12,13,14^ The eligibility criteria are shown in Table 1.

**TABLE 1:** Eligibility criteria for the qualitative evidence synthesis.

Question element	Eligibility criteria
Sample (participants)	Considering the importance of a child-health focus, we identified key stakeholder groups whose perspectives we focused on for each of the phenomena of interest, as follows: Acceptability: children and caregivers – receiving or affected by the intervention Feasibility: children and caregivers, intervention providers – receiving, delivering or affected by the intervention Equity: children and caregivers – receiving or affected by the intervention Studies that included participants from key stakeholder groups as well as from other stakeholder groups were only included if data representing the perspectives of key stakeholder groups could be extracted.
Phenomena of interest	Acceptability, feasibility and equity implications (criteria as outlined the GRADE Evidence-to-Decision Framework) of: nutritional supplementation interventions and strategies (single and/or multicomponent) delivered within an entire community or a large part of a community, aimed at preventing wasting in infants and young children (< 5 years of age); and the delivery of these interventions, specifically, blanket, targeted and coverage Our interpretations of these phenomena were framed to align with their conceptualisation as three of the criteria in the GRADE EtD framework: Acceptability: ‘the extent to which that intervention is considered to be reasonable among those receiving, delivering or affected by the intervention’.^13^ Feasibility of an intervention: ‘the likelihood that it can be properly carried out or implemented in a given context’.^13^ Equity: potential impacts on equity should be evaluated ‘in relation to specific characteristics that are likely to be associated with disadvantage in relation to the question being addressed’.^14^
Design	Qualitative designs and methods for data collection and analysis
Evaluation	Perspectives (views, experiences, opinions, attitudes, perceptions)
Research type	Full-text primary studies or evaluations published in English from 1990 onwards. Both studies using qualitative study designs and mixed-methods designs were eligible, provided the latter reported extractable qualitative data that were collected and analysed qualitatively, regardless of whether they were conducted alongside studies of intervention effectiveness or not. Grey literature (e.g. research reports, programme descriptions and evaluations not published by a commercial publisher) as well as formative research were also eligible. We excluded studies that collected data using qualitative methods but did not analyse these data using qualitative analysis methods, publications that did not report on primary research, studies for which no full-text publications were available or published in a language other than English, preprint publications that have not been peer-reviewed and accepted for publication and formative research used as part of an iterative process of refining the intervention (i.e. not associated with an intervention implemented in the real-world setting). We also excluded studies that were considered ‘hypothetical’, that is, studies where participants had not received or provided preventative interventions to reduce childhood wasting.
Settings	Studies conducted in any country or setting.
Interventions	The scope of eligible interventions in this QES was originally aligned with the scope of eligible interventions included in the systematic review about the effects of interventions for the prevention of wasting in infants and young children also commissioned for this guideline.^15^ It included interventions and strategies (single and/or multicomponent), delivered within an entire community or a large part of a community, and aimed at preventing wasting in infants and children. Following recategorisation of nutritional supplementation interventions by the WHO, the following interventions and strategies directed at infants, children and/or caregivers and mothers (including during pregnancy) to prevent wasting were eligible: Food supplementation with alternative foods Nutritional supplementation with SQ-LNS Nutritional supplementation with RUSF Nutritional supplementation with FBF Micronutrient supplementation (such as MMP). ‘Wasting prevention’ included primary prevention (prevention of any wasting), secondary prevention (prevention of worsening of wasting, i.e., from moderate to severe) and prevention of relapse to wasting in children who have been treated for wasting, across eligible intervention contexts and along a ‘continuum’ of severity. Studies addressing only treatment interventions for severe wasting were excluded, as were studies aimed at preventing or treating stunting. Studies on both preventive and treatment interventions were only included if relevant data about the preventive intervention(s) could be extracted separately. For each phenomenon, we included the relevant perspectives of key stakeholders about the eligible intervention as well as the delivery of these interventions, specifically: ‘blanket’ delivery (interventions delivered to all persons or households in an affected population or catchment area, for example, an entire community, neighbourhood, city, state or nation, without targeting specific individuals or subgroups) targeted or selected delivery (interventions delivered according to specified criteria aimed at only targeting specific individuals or subgroups that met certain anthropometric, clinical or social criteria used as proxies for being nutritionally at risk) coverage (the proportion of the target population reached by an intervention).

Note: Please see the full reference list of the article Brand AS, Visser ME, Kallon II, Van Wyk SS, Rohwer AC. Acceptability, feasibility and equity implications of nutritional supplementation interventions for the prevention of wasting in infants and young children: A rapid qualitative evidence synthesis. Afr J Prm Health Care Fam Med. 2026;18(1), a5137. https://doi.org/10.4102/phcfm.v18i1.5137, for more information.

EtD, evidence-to-decision; FBF, fortified blended food; GRADE, Grading of Recommendations, Assessment, Development and Evaluations; MMP, multiple micronutrient powder; QES, qualitative evidence synthesis; RUSF, ready-to-use supplementary food; SQ-LNS, small-quantity lipid-based nutrient supplement.

### Search methods for identification of studies

We designed a pre-planned, comprehensive search strategy together with a Cochrane information specialist and content experts working in IYC wasting (see Online Appendix 1 – Table 1). Because of time constraints and the need for a rapid review, we only conducted a systematic search in one electronic database, namely MEDLINE (PubMed), which is one of the largest searchable biomedical databases, on 13 June 2022. No language restrictions were applied. As we only searched one database, we also undertook targeted searching to identify eligible ‘trial siblings’ of relevant effectiveness trials included in the reference list of the commissioned systematic review on interventions for the prevention of wasting in infants and young children.^15^ When considering qualitative studies in a QES about intervention effects linked to an intervention review, two types of qualitative studies are available: those that collect data from the same participants as effectiveness trials in the review, known as ‘trial siblings’, and those that address relevant issues about the intervention, but as separate items of research and not connected to relevant effectiveness trials.^16^ Both can provide useful information, with trial sibling studies being closer to relevant effectiveness trials in terms of their precise contexts, and non-sibling studies possibly contributing perspectives not present in the trials.^17,18^ Trial siblings and articles reporting intervention effectiveness as well as phenomenological qualitative data were considered as *direct* sources of evidence regarding the interventions evaluated in the effectiveness review. Non-sibling studies were considered *indirect* sources of evidence regarding interventions evaluated in the effectiveness review. In consultation with the WHO, we also contacted experts from key stakeholder organisations to request potentially relevant reports and evaluations these organisations may be aware of.

### Selection of studies

Titles and abstracts of studies identified through the searches were divided among the reviewers for screening against eligibility criteria using the Screenatron tool from Bond University’s Systematic Review Accelerator.^19^ Where a single reviewer was uncertain about potential eligibility of titles and abstracts, they consulted with a second reviewer. As a result of a lack of clarity about the exact nature of interventions or phenomena in many titles and abstracts, we screened over-inclusively at the title and abstract stage and introduced an additional ‘pre-screening’ step to assess preliminary eligibility of full texts relating to the intervention prior to comprehensive full-text screening. This was conducted in Microsoft^®^ Excel and represented a necessary deviation from our *a priori* protocol, given large yields and the rapid nature of the work. Full-text articles that were pre-screened as ‘potentially eligible’ were assessed by at least two reviewers, with a broader group discussion, if needed, to decide on eligibility for inclusion in the review. Approximately one quarter of the comprehensive full-text screening and contextual data extraction process was performed by the whole author team to ensure calibration. Studies where full text(s) did not provide enough information to enable a decision about eligibility were listed under ‘studies awaiting classification’. We include in this report a Preferred Reporting Items for Systematic Reviews and Meta-Analyses (PRISMA) flow diagram summarising the overall results of our search, as well as the screening process and inclusion/exclusion of studies. We collated multiple publications describing the same study and using the same sample and methodology, thereby making the specific study the unit of analysis in our review.

### Data extraction, analysis and synthesis

Data extraction, analysis and synthesis were performed iteratively as needed using the best-fit framework synthesis method, as this approach saves time and works well where there is a clear framework to support the synthesis.^10,11^ We identified suitable frameworks for acceptability, feasibility and equity through snowball searches.^20,21,22,23^ We used the constructs within each of these to generate an *a priori* coding framework for the synthesis, comprising the constructs for each phenomenon, their definitions and codes (Online Appendix 1 – Table 2). We familiarised ourselves with the data by reading and re-reading the individual primary studies included in the analysis. As part of the indexing stage, we firstly extracted descriptive ‘contextual’ details for each eligible study and captured these data in Microsoft^®^ Excel. Secondly, we coded data deductively according to the pre-specified coding framework (see Online Appendix 1 – Table 2), using ATLAS.ti Windows (Version 22.1.5.0). A single author (A.S.B., M.E.V., I.I.K. or S.S.v.W.) coded the data, which included quotes from participants, author interpretations, themes and sub-themes, new theory or observational excerpts. Although we allowed for inductive coding as per the best-fit framework synthesis method, all data fit into the constructs of the existing frameworks. Thirdly we organised quotations according to intervention categories and constructs within each phenomenon of interest. At least three members of the author team met to discuss the codes and quotations for each construct within separate intervention categories and identified and synthesised emerging themes in an iterative process. Finally, we presented findings that aim to render this rapid QES ‘fit for purpose’.^24^ To this end, we presented findings separately for each of the phenomena of interest and specific intervention, which are also the three criteria in the EtD framework (namely acceptability, feasibility and equity). We also identified whether findings were informed by ‘direct’ and ‘indirect’ evidence. As far as possible and relevant, we sought to present and summarise findings to align with the questions for judgements for the three criteria in the GRADE EtD framework in GRADEPro.^13,14^

**TABLE 2:** Characteristics of included studies reporting phenomena of interest around nutritional supplementation interventions.

Study	Data collection	Data analysis	Phenomenon	Participants	Intervention delivery	Country and setting	Description of intervention	Description of delivery approach
1. **Infant and young child supplementation**								
a. **Interventions to supplement complementary foods**								
**Alternative foods**								
Dewi Satiawati 2018^41^ (indirect evidence)	Individual interviews	Thematic	Acceptability, feasibility, equity	Caregivers (*n* = 16)	Targeted	Indonesia, urban (Denpasar city)	Formula milk and toddlers’ rusk	Provided for a period of 90 days or until recovery is medically certified.
Waters 2018^59^ (indirect evidence)	Interviews, focus group discussions and structured observations	Thematic	Acceptability	Caregivers (number not reported)	Blanket	Ecuador, rural communities	One egg per day per infant	Weekly supply provided for six months.
Small-quantity lipid-based nutrient supplement (SQ-LNS)								
Ashorn 2015^38^ (direct evidence)	In-depth interviews and questionnaires	Framework	Acceptability, equity	Caregivers (*n* = 30)	Blanket	Malawi, rural	Lipid-based nutrient supplement (SQ-LNS) (iLiNS project supplement)	Two-week supply provided – home delivery or at health centre (varying quantities LNS/day ranging from 10 g/day – 40 g/day).
Lesorogol 2015^49^ (indirect evidence)	Interviews, focus groups and observations	Framework	Acceptability, feasibility	Caregivers (*n* = 29)	Blanket	Haiti, poor urban area with many rural migrants	Lipid-based nutrient supplements (SQ-LNS)	Hospital clinic provided monthly for three or six months; LNS providing 108 kcal per day.
Paul 2012^56^ (direct evidence)	Individual interviews	Thematic	Acceptability, feasibility	Caregivers (*n* = 16)	Blanket	Zimbabwe, rural	Lipid-based nutrient supplement (SQ-LNS) (Nutributter)	Nine-day supply provided (20 g per day providing 452 kJ per day).
Rothman 2015^57^ (direct evidence)	Focus group discussions and interviews	Thematic	Acceptability	Caregivers (*n* = 38)	Blanket	South Africa, peri-urban	Small-quantity lipid-based nutrient supplement (SQ-LNS)	Thirteen-day supply provided (20-g per day SQ-LNS).
**Ready-to-use supplementary food (RUSF)**								
Cohuet 2010^38^ (direct evidence)	Focus groups, individual interviews and structured questionnaire	Qualitative content	Acceptability, equity	Caregivers (*n* = 128)	Targeted	Niger, rural villages	Ready-to-Use Supplementary Food (RUSF) (Plumpy’doz and Supplementary’Plumpy)	Monthly supply provided (46 g/day and one 92 g sachet/day).
Iuel-Brockdorf 2015^42^ (indirect evidence)	Interviews, focus group discussions and observations	Qualitative content	Acceptability	Caregivers (*n* = 38)	Targeted	Burkina Faso, rural	Lipid-based nutrient supplements (LQ-LNS) with varying quantities of milk and soy isolate/dehulled soy	Three-day supply provided (92 g LNS/day, providing 500 kcal/day).
Iuel-Brockdorf 2016^43^ (indirect evidence)	Observations and interviews, survey and anthropometry	Qualitative content	Acceptability, equity	Caregivers (*n* = 71)	Targeted	Burkina Faso, rural	Lipid-based nutrient supplements (LQ-LNS) with varying quantities of milk and soy isolate	Provided by local health centres (92 g LNS/day, providing 500 kcal/day).
Iuel-Brockdorf 2017^44^ (indirect evidence)	Home visits, interviews and focus group discussions, questionnaires	Qualitative content	Acceptability, equity	Caregivers (*n* = 71)	Targeted	Burkina Faso, rural	Lipid-based nutrient supplements (LQ-LNS) with varying quantities of milk and soy isolate	Provided by local health centres (92 g LNS/day, providing 500 kcal/day).
Langlois 2020^48^ (indirect evidence)	Interviews, focus group discussions and in-home observations	Thematic	Acceptability, feasibility, equity	Caregivers (*n* = 1654)	Blanket	Burkina Faso	Ready-to-use supplemental food (RUSF)	Monthly supply provided by distribution centres, providing 500 kcal/day.
Marquer 2020^51^ (direct evidence)	Focus group discussions and in-depth interviews	Thematic	Acceptability, feasibility, equity	Caregivers (*n* = 114)	Blanket	Niger, villages	Ready-to-use-supplementary food (RUSF-Supplementary Plumpy) and lipid-based Nutrient Supplement Medium Quantity (LNS-MQ, Plumpy’Doz)	Monthly supply provided (92 g-sachet RUSF per day and 325 g pot LNS-MQ/week, given as 46 g per day).
Muraya 2017^54^ (indirect evidence)	Individual and group interviews and focus group discussions	Framework	Feasibility, equity	Caregivers (*n* = 15)	Targeted	Coastal Kenya, rural sub-location	Ready-to-use supplementary food (RUSF-Plumpy-Sup)	Two-week supply provided by health facilities.
**Fortified blended food (FBF)**								
Iuel-Brockdorf 2015^44^ (indirect evidence)	Interviews, focus group discussions and observations	Qualitative content	Acceptability	Caregivers (*n* = 38)	Targeted	Burkina Faso, rural	Corn-soy blended flours (CSB) with varying quantities of milk and soy isolate/dehulled soy	Three-day supply provided (120 g CSB/day, providing 500 kcal/day).
Iuel-Brockdorf 2016^43^ (indirect evidence)	Observations and interviews, survey and anthropometry	Qualitative content	Acceptability, equity	Caregivers (*n* = 71)	Targeted	Burkina Faso, rural	Corn-soy blended flours (CSB) with varying quantities of milk and soy isolate	Provided by local health centres (120 g CSB/day, providing 500 kcal/day).
Iuel-Brockdorf 2017^44^ (indirect evidence)	Home visits, interviews and focus group discussions, questionnaires	Qualitative content	Acceptability, equity	Caregivers (*n* = 71)	Targeted	Burkina Faso, rural	Corn-soy blended flours (CSB) with varying quantities of milk and soy isolate	Provided by local health centres (120 g CSB/day, providing 500 kcal/day).
Langlois 2020^48^ (indirect evidence)	Interviews, focus group discussions and in-home observations	Thematic	Acceptability, feasibility, equity	Caregivers (*n* = 1654)	Blanket	Burkina Faso	Corn-soy blend plus oil, corn-soy whey blend plus oil or Super Cereal Plus	Monthly supply provided by distribution centres (varying daily rations, each providing 500 kcal/day).
Marquer 2020^51^ (direct evidence)	Focus group discussions and in-depth interviews	Thematic	Acceptability, feasibility, equity	Caregivers (*n* = 114)	Blanket	Niger, villages	Corn-soy blend (CSB-Super Cereal Plus)	Monthly supply provided (200 g CSB per day).
Muraya 2017^54^ (indirect evidence)	Individual and group interviews and focus group discussions	Framework	Feasibility, equity	Caregivers (*n* = 15)	Targeted	Coastal Kenya, rural sub-location	Fortified corn-soy blend flour mixed with vegetable oil	Two-week supply provided by health facilities.
b. **Child micronutrient supplementation**								
Creed Kanashiro 2016^39^ (indirect evidence)	Interviews and observations	Thematic	Acceptability, feasibility, equity	Caregivers (*n* = 35) and intervention providers (*n* = 11)	Blanket	Peru, peri-urban and rural	Multiple micronutrient powder	Monthly or bi-monthly supply provided by local health facility (one sachet every other day for 6 months, followed by a rest period of 6 months).
Jefferds 2010^45^ (direct evidence)	Focus group discussion	Qualitative content	Acceptability, feasibility	Caregivers (*n* = 14)	Blanket	Western Kenya, rural	Multiple micronutrient powder (Sprinkles)	Thirty-day supply provided (1-g sachet per day).
Loechl 2009^50^ (indirect evidence)	Focus groups, individual interviews, structured observations, exit interviews, checks of ration cards and survey data	Thematic	Acceptability, feasibility	Caregivers (between *n* = 24 and *n* = 32) and intervention providers (between *n* = 35 and *n* = 70)	Blanket	Haiti, rural	Multiple micronutrient powder (Sprinkles)	Two-monthly supply provided at food distribution points; equivalent to one sachet per day.
Mridha 2012^53^ technical report (direct evidence)	Focus group discussions, test feeding and home use	Thematic	Acceptability, feasibility, equity	Caregivers (*n* = 20) and intervention providers (*n* = 2)	Blanket	Bangladesh, urban and rural	Multiple micronutrient powder (Monimix)	Fourteen-day supply provided (1-g sachet per day).
2. **Maternal supplementation**								
a. **Maternal food supplementation**								
**Alternative foods**								
Stevens 2018^58^ (direct evidence)	Semi-structured questionnaire (quantitative and qualitative)	Thematic	Acceptability, feasibility	Caregivers (*n* = 10)	Targeted	Bangladesh, rural	Locally produced protein–energy food-based supplement	Fifteen-day supply provided twice by community nutrition volunteers (one packet/day).
**Small-quantity lipid-based nutrient supplement (SQ-LNS)**								
Harding 2017^41^ (direct evidence)	Interviews and questionnaire	Constant comparison	Acceptability, feasibility	Caregivers (*n* = 16) and intervention providers (*n* = 8)	Blanket	Bangladesh, rural	Lipid-based nutrient supplement (SQ-LNS)	Monthly supply provided by community health workers/ volunteers; LNS provided at 20 g/day for the equivalent of 118 kcal.
**Ready-to-use supplementary food (RUSF)**								
Chunda-Liyoka 2020^37^ (indirect evidence)	Focus group discussion	Qualitative content	Acceptability	Caregivers (*n* = 27)	Blanket	Zambia, urban	Ready-to-use supplemental food (RUSF) containing fish oil (DHA)	Not reported
**Fortified blended food (FBF)**								
No studies identified								
b. **Maternal micronutrient supplementation**								
Noznesky 2012^55^ situation analysis (indirect evidence)	Individual interviews	Thematic	Feasibility	Intervention providers (*n* = 48)	Blanket	Eastern India, urban and rural	Iron and folic acid (IFA) supplement	Provision of 100 tablets (100 mg ferrous sulphate and 0.5 mg folic acid) during pregnancy.
Williams 2020^60^ (indirect evidence)	Focus group discussions, in-depth interviews and key informant interviews	Thematic	Acceptability, feasibility, equity	Caregivers (*n* = 28) and intervention providers (*n* = 14)	Blanket	India, villages	Iron and folic acid (IFA) supplement	Government- mandated provision of 100 IFA tablets during pregnancy.
3. **Maternal and child supplementation**								
a. **Maternal and child food supplementation**								
**Alternative foods**								
Mennillo 2014^52^ (indirect evidence)	Individual interviews	Thematic (immersion/crystallisation method)	Feasibility, equity	Intervention providers (*n* = 8)	Blanket	Honduras, remote, rural	Fortified oatmeal, eggs, and wheat	Monthly supply provided.
Small-quantity lipid-based nutrient supplement (SQ-LNS)								
Klevor 2016^48^ (direct evidence)	Questionnaire (adherence), in-depth interviews (acceptability)	Thematic	Acceptability, equity	Caregivers (*n* = 62)	Blanket	Ghana, semi-urban; Malawi, rural	Small-quantity lipid-based nutrient supplement (SQ-LNS)	Two-week supply provided during home visits (20 g-sachet/day).
Mridha 2012^53^ technical report (direct evidence)	Focus group discussions, test feeding and home use	Thematic	Acceptability, feasibility, equity	Caregivers (*n* = 20) and intervention providers (*n* = 2)	Blanket	Bangladesh, urban and rural	Lipid-based nutrient supplement (SQ-LNS – regular, cumin- or cardamom-flavoured)	Fourteen-day supply provided (20 g-sachet/day for PLW; 10-g sachet twice a day for IYC; both for the equivalent of 118 kcal).
**Ready-to-use supplementary food (RUSF)**								
No studies identified								
**Fortified blended food (FBF)**								
No studies identified								
b. **Maternal and child micronutrient supplementation**								
Kodish 2011^47^ (indirect evidence)	Interviews, observations and focus group discussions	Grounded theory	Acceptability, feasibility, equity	Caregivers (*n* = 45) and intervention providers (*n* = 14)	Blanket	North-western Kenya, refugee camp	Multiple micronutrient powder	Monthly supply provided by food distribution centres (one sachet/day).

Note: Please see the full reference list of the article Brand AS, Visser ME, Kallon II, Van Wyk SS, Rohwer AC. Acceptability, feasibility and equity implications of nutritional supplementation interventions for the prevention of wasting in infants and young children: A rapid qualitative evidence synthesis. Afr J Prm Health Care Fam Med. 2026;18(1), a5137. https://doi.org/10.4102/phcfm.v18i1.5137, for more information.

CSB, corn-soy blend; DHA, docosahexaenoic acid; IFA, iron and folic acid; IYC, infants and young children; LNS, lipid-based nutrient supplement; LQ, large quantity; MQ, medium quantity; NA, not applicable; PLW, pregnant and lactating women; RUSF, ready-to-use supplementary food; RUTF, ready-to-use therapeutic food; SQ, small-quantity.

### Assessing the methodological limitations of included studies

A single reviewer (A.S.B., M.E.V., I.I.K. or S.S.v.W.) assessed methodological limitations for each study using an adapted version of the Critical Skills Appraisal Programme (CASP) tool.^25,26^ For each included study, a single reviewer (A.S.B., M.E.V., I.I.K. or S.S.v.W.) applied the CASP criteria as they pertain to the validity of results, what the results are and whether they will help locally. Any uncertainties were discussed with the review team. We did not use our assessment of methodological limitations to exclude studies.

### Assessment of confidence in review findings

Given the timeframe and rapid nature of this response, we did not assess the confidence in each review finding using GRADE-CERQual (Confidence in the Evidence from Reviews of Qualitative research).

### Reviewer reflexivity

Any type of qualitative research is subjective and can be influenced by personal beliefs, experiences and backgrounds. We convened regular meetings throughout the review process to reflect on personal biases and judgements linked to selection of studies, new codes and emerging themes. None of the authors are members of the GDG; however, we liaised with the WHO technical team and methodologists for the guideline to ensure that the QES is fit-for-purpose. All reviewers are employed by the Centre for Evidence-based Health Care (CEBHC), Stellenbosch University, Cape Town, South Africa, except for I.I.K., who is a post-doctoral fellow at the CEBHC. Authors are involved with quantitative and/or qualitative research synthesis and primary studies and have academic and/or clinical backgrounds: nursing and midwifery, and clinical epidemiology (A.C.R.), public health and clinical epidemiology (A.S.B.), clinical nutrition, public health and clinical epidemiology (M.E.V.), medicine and public health (S.S.v.W.) and public health (I.I.K.). M.E.V. has been involved with programmes that included treatment and prevention of wasting. As a clinician, S.S.v.W. has treated wasted children with acute and chronic illnesses as well as children with kwashiorkor.

## Review findings

### Results of the searches

**FIGURE 1:**
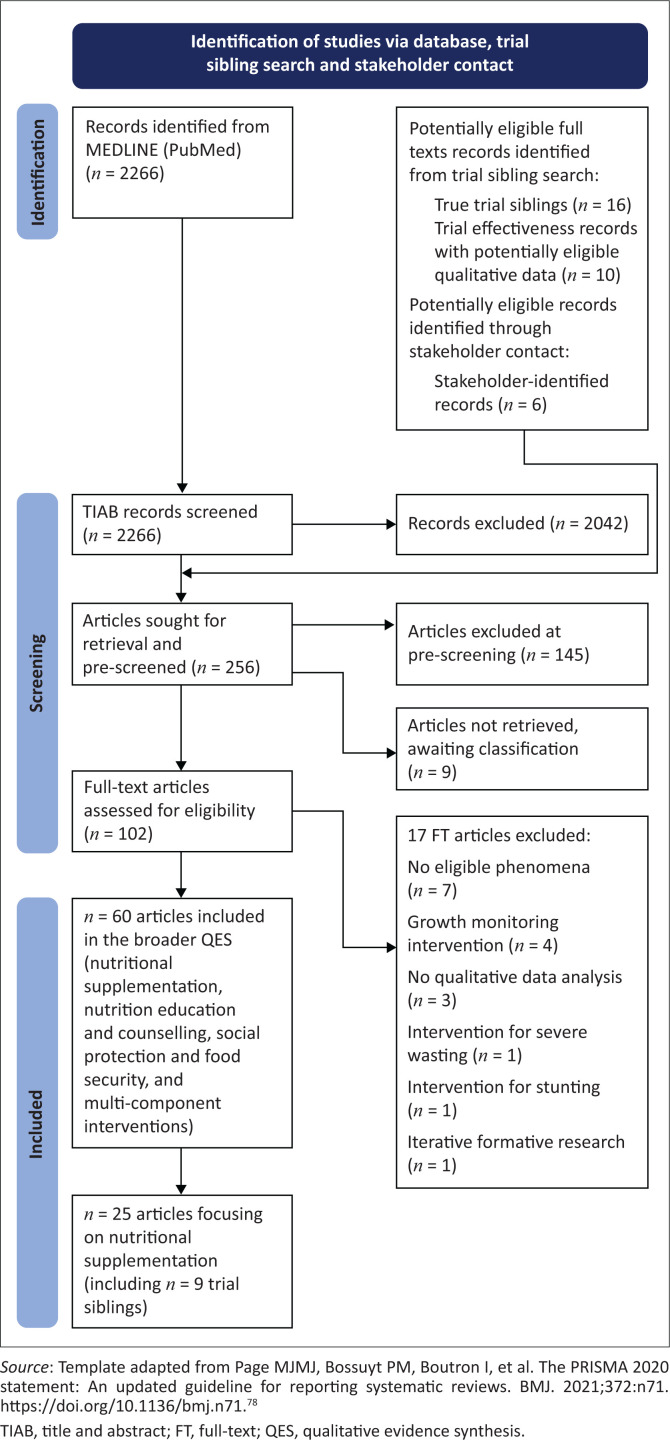
Preferred Reporting Items for Systematic Reviews and Meta-Analyses (PRISMA) diagram for the stage 1 search for eligible systematic reviews.

We screened the titles and abstracts of 2266 records identified through searching. Following the inclusion of 26 potentially eligible full texts from the trial sibling search and six potentially eligible full texts from contact with stakeholders identified by the WHO, we pre-screened 256 full texts to determine whether the nature of the intervention and phenomena appeared eligible. We could not locate nine records.^27,28,29,30,31,32,33,34,35^ We placed these under awaiting classification and excluded 145 records at the pre-screening stage. We screened 102 full texts against our full eligibility criteria. A total of 85 full text articles were eligible across broad intervention categories, some of which are not included in the scope of this article. Sixteen of these articles were trial siblings, and two articles were articles on intervention effectiveness that also reported relevant qualitative information. A total of 25 articles reporting on 32 nutritional supplementation interventions were included in this article.^36,37,38,39,40,41,42,43,44,45,46,47,48,49,50,51,52,53,54,55,56,57,58,59,60^ We excluded 17 full-text records, with reasons for exclusion per study summarised in Online Appendix 1 – Table 3.^61,62,63,64,65,66,67,68,69,70,71,72,73,74,75,76,77^ The study selection flowchart is available in Figure 1.

**TABLE 3:** A summary of themes for nutritional supplementation.

Qualitative evidence for intervention categories†,‡	Phenomenon	Themes	Perspectives on coverage §
**Infant and child supplementation**			
**Complementary food**			
**Alternative foods** Direct evidence: no articles Indirect evidence (*n* = 2): Ecuador (provision of one egg a day); Indonesia (formula milk and toddlers’ rusks)	Acceptability	**Theme 1.1:** Alternative foods are mostly perceived to be good for infants and children, but acceptability is affected by familiarity (indirect evidence)	No
	Feasibility	**Theme 1.2:** Caregivers not understanding the rationale for providing alternative foods as well as discomfort about receiving support may act as barriers to uptake, but this can be counteracted by motivation from healthcare workers (indirect evidence)	No
	Equity	No themes emerging	No
**Small-quantity lipid-based nutrient supplement** Direct evidence (*n* = 4): Haiti (SQ-LNS); Malawi (SQ-LNS); South Africa (SQ-LNS); Zimbabwe (SQ-LNS) Indirect evidence: no articles	Acceptability	**Theme 2.1:** SQ-LNS is mostly considered to be good for child well-being and convenient, and children enjoy them (direct and indirect evidence)	No
	Feasibility	**Theme 2.2:** Caregivers do not always have control over providing SQ-LNS for infants and children in the way that it is intended (indirect evidence)	No
	Equity	No themes emerging	No
**Ready-to-use supplementary food** Direct evidence (*n* = 1): Niger (RUSF) Indirect evidence (*n* = 6): Burkina Faso (LQ-LNS, RUSF); Kenya (RUSF); Niger (RUSF, MQ-LNS)	Acceptability	**Theme 3.1:** RUSF is mostly considered to be good for child well-being and convenient, and children enjoy it (direct and indirect evidence)	No
	Feasibility	**Theme 3.2:** Information and education may facilitate RUSF use, while social factors and poor sanitation may be barriers to implementation (direct and indirect evidence)	No
	Equity	**Theme 3.3:** Caregivers do not always have control over providing RUSF for infants and children in the intended amount because of sharing practices (direct and indirect evidence)	No
**Fortified blended foods** Direct evidence: no articles Indirect evidence (*n* = 6): Burkina Faso (CSB, CSB plus oil, CSWB plus oil, Super Cereal Plus); Kenya (CSB with oil); Niger (CSB-Super Cereal Plus)	Acceptability	**Theme 4.1:** FBFs are thought to be good for children and are considered medicines by some, but sensory attributes and burden of preparation may affect acceptability (direct and indirect evidence)	No
	Feasibility	**Theme 4.2:** Education may facilitate FBF use while social factors, taste and quality, as well as limited sanitation may be barriers to implementation (direct and indirect evidence)	No
	Equity	**Theme 4.3:** Caregivers do not always have control over providing FBF to intended infants and children only (direct and indirect evidence)	No
**Micronutrient supplementation** Direct evidence (*n* = 2): Bangladesh (MMP); Kenya (MMP) Indirect evidence (*n* = 2): Haiti, Peru	Acceptability	**Theme 5.1:** If adequately informed, caregivers mostly consider child micronutrient supplementation to benefit child well-being and be convenient (direct and indirect evidence)	Yes
	Feasibility	**Theme 5.2:** Misconceptions of intervention providers and poor communication hinders implementation of child MNS (direct and indirect evidence)	No
	Equity	No themes emerging	No
**Maternal supplementation**			
**Food supplementation**			
**Alternative foods** Direct evidence (*n* = 1): Bangladesh (protein–energy food-based supplement) Indirect evidence: no articles	Acceptability	**Theme 6.1:** Perspectives on the tolerability and health benefits of alternative food for mothers were mixed (direct evidence)	No
	Feasibility	No themes emerging	No
	Equity	No perspectives found	No
**Small-quantity lipid-based nutrient supplement** Direct evidence (*n* = 1): Bangladesh (SQ-LNS) Indirect evidence: no articles	Acceptability	**Theme 7.1:** Acceptability of and adherence to SQ-LNS for mothers was affected by sensory experiences, perceptions on side effects and beliefs about health benefit (direct evidence)	No
	Feasibility	**Theme 7.2:** Mothers do not always trust unfamiliar or foreign SQ-LNS food products, which impacts adherence (direct and indirect evidence)	No
	Equity	No perspectives found	No
**Ready-to-use supplementary food** Direct evidence: no articles Indirect evidence (*n* = 1): Zambia (RUSF with DHA)	Acceptability	**Theme 8.1:** Acceptability of RUSF for mothers was affected by sensory experiences and perceptions on side effects although beliefs about health benefits improved adherence (indirect evidence)	No
	Feasibility	No perspectives found	No
	Equity	No perspectives found	No
**Fortified blended food** Direct evidence: no articles Indirect evidence: no articles	Acceptability	No articles	No
	Feasibility	No articles	No
	Equity	No articles	No
**Micronutrient supplementation** Direct evidence: no articles Indirect evidence (*n* = 2): India (IFA supplements)	Acceptability	**Theme 9.1:** Uptake of maternal MNS is poor because of its taste, side effects, competing priorities and misconceptions around the intervention (indirect evidence)	No
	Feasibility	**Theme 9.2:** A lack of prioritisation of maternal malnutrition affects feasibility of providing maternal MNS (indirect evidence)	No
	Equity	**Theme 9.3:** Differential access to maternal MNS caused inequity (indirect evidence)	No
**Maternal and child supplementation**			
**Food supplementation**			
**Alternative foods** Direct evidence: no articles Indirect evidence (*n* = 1): Honduras (fortified oatmeal, eggs and wheat)	Acceptability	No perspectives found	No
	Feasibility	**Theme 10.1:** Community involvement in the production of alternative foods for mothers and children can ensure sustainability (indirect evidence)	No
	Equity	**Theme 10.2:** Intra-household food distribution is a cultural practice that affects intervention adherence to alternative food for mothers and children (indirect evidence)	No
**Small-quantity lipid-based nutrient supplement** Direct evidence (*n* = 2): Bangladesh (SQ-LNS); Ghana and Malawi (SQ-LNS) Indirect evidence: no articles	Acceptability	**Theme 11.1:** PLW may not always tolerate SQ-LNS, but most children tend to enjoy it (direct evidence) **Theme 11.2:** Experiences of self and others influence the uptake of SQ-LNS for mothers and children (direct evidence) **Theme 11.3:** People interact differently with SQ-LNS for mothers and children based on whether they believe it to be medicine or not (direct evidence)	Yes
	Feasibility	No themes emerging	Yes
	Equity	**Theme 11.4:** Gender roles and cultural sharing practices may result in SQ-LNS for mothers and children not reaching its intended beneficiaries (direct evidence)	No
**Ready-to-use supplementary food** Direct evidence: no articles Indirect evidence: no articles	Acceptability	No articles	No
	Feasibility	No articles	No
	Equity	No articles	No
**Fortified blended food** Direct evidence: no articles Indirect evidence: no articles	Acceptability	No articles	No
	Feasibility	No articles	No
	Equity	No articles	No
**Micronutrient supplementation** Direct evidence: no articles Indirect evidence (*n* = 1): Kenya (MMP)	Acceptability	**Theme 12.1:** Information about MMP for mothers and children, and its purpose, is important for acceptance (indirect evidence)	Yes
	Feasibility	No perspectives found	No
	Equity	**Theme 12.2:** A lack of information and cultural/religious sensitivity may exacerbate inequity in the provision of maternal and child MNS (indirect evidence)	No

CSB, corn-soy blend; CSWB, corn-soy whey blend; DHA, docosahexaenoic acid; FBF, fortified blended food; IFA, iron-folic acid; LNS, lipid-based nutritional supplement; LQ, large-quantity; MMP, multiple micronutrient powder; MNS, maternal micronutrient supplementation; MQ, medium-quantity; PLW, pregnant and lactating women; RUSF, ready-to-use supplementary food; SQ, small-quantity.

† , Direct evidence is defined as trial siblings (articles that collect data from the same participants as effectiveness trials) and articles reporting interventions effectiveness as well as phenomenological qualitative data;

‡ , Indirect evidence is defined as non-trial sibling articles;

§ , Coverage is defined as the proportion of the target population reached by an intervention.

### Description of included studies

Characteristics of the included studies are provided in Table 2, and the methodological limitations of included studies are presented in Online Appendix 1 – Table 4.

Perspectives on acceptability, feasibility and equity of nutritional supplementation interventions.

The findings related to nutritional supplementation for the prevention of wasting, as well as the availability of information related to coverage, are summarised in the summary table of findings presented in Table 3.

#### Infant and child supplementation: Complementary foods

**Theme 1.1: *Alternative foods* are mostly perceived to be good for infant and young child, but acceptability is affected by familiarity (indirect evidence):** Caregivers felt that IYC had improved health, physical and cognitive development when provided with alternative foods that are familiar to them. As a result of this experience, caregivers perceived these interventions to be effective. Some caregivers, however, felt that some foods, such as eggs, can be harmful for IYC. Alternative foods that beneficiaries were unfamiliar with were less acceptable as children often refused them, and caregivers preferred the formula milks they knew.

**Theme 1.2: Caregivers not understanding the rationale for providing *alternative* foods as well as discomfort about receiving support may act as barriers to uptake, but this can be counteracted by motivation from healthcare workers (indirect evidence):** Perspectives from caregivers suggested that many were unwilling to accept that their child is moderately wasted, with one caregiver stating that the child is ‘just naturally small’. In cases where caregivers did acknowledge their child’s condition, many attributed this to witchcraft and consequently could not see how the foods would remedy the situation. These misconceptions presented as barriers to uptake, as many of these caregivers refused to provide the alternative foods to their children. Caregivers expressed a preference for alternative foods they are familiar with, such as age-specific, commercially available milks that are highly marketed. They also reported feeling ashamed and stigmatised by the provision of alternative foods at home, as they felt that social perception would be that they have a sick child or are unable to care for their children. However, second-order constructs indicated that caregivers found fetching alternative foods from health centres burdensome and objected to this. These factors may present as barriers to implementation. It was highlighted in second-order constructs by researchers that healthcare workers (HCWs) who understand the barriers to effecting behavioural change can play an important role as facilitators by motivating and stimulating the participation of caregivers.

**Theme 2.1: Small-quantity lipid-based nutrient supplement is mostly considered to be good for child well-being and convenient, and children enjoy them (direct and indirect evidence):** Caregivers felt that the nutritional supplements improved the appetite, vitality, health, activity, physical and cognitive development of their children. They also did not experience feeding children with SQ-LNS to be inconvenient and found preparation easy. Most caregivers reported that children enjoy the taste of SQ-LNS; some children disliked it initially, but they grew to enjoy it.

**Theme 2.2: Caregivers do not always have control over providing small-quantity lipid-based nutrient supplement for infant and young child in the way that it is intended (indirect evidence):** Some caregivers reported practices that may cause target children not to receive SQ-LNS as intended, potentially causing a barrier to effective implementation. In some cases, SQ-LNS was shared because other children in the household also enjoyed the taste, with other caregivers reporting that they locked the supplement away so other household members could not access it. Not all caregivers gave SQ-LNS alongside other complementary food as instructed because of a lack of resources. In addition, certain caregivers raised concerns about providing the SQ-LNS in a hygienic manner because of poor or non-existent sanitation facilities and a lack of clean water.

**Theme 3.1: Ready-to-use supplementary food is mostly considered to be good for child well-being and convenient, and children enjoy it (direct and indirect evidence):** There was high acceptability for RUSF among caregivers as they experienced it as convenient and relieving the financial burden on the household. Caregivers felt that the nutritional supplements improved the appetite, vitality, health, activity, physical and cognitive development of their children. Some caregivers believed that the RUSF is medicine and Western food of higher effectiveness than those found locally; however, the uncertainty around its provenance caused distrust of the product for some. Because of its perceived effectiveness and the perception of RUSF being medicine, most caregivers did not share it with other members of the household, although some did indicate that they gave small quantities to other children. Some breastfeeding mothers also reported consuming some of the RUSF intended for their children as they believed it guaranteed their milk supply. Most caregivers reported that children enjoy the taste of RUSF; some children disliked it initially, but they grew to enjoy it. Many caregivers reported that they mixed RUSF with other foods initially so children would eat it.

**Theme 3.2: Information and education may facilitate ready-to-use supplementary fooduse, while social factors and poor sanitation may be barriers to implementation (direct and indirect evidence):** The need for information and education sessions as facilitators to implementation was highlighted, with researchers noting the impact of these on the consumption of the product. There was an interaction of gender roles, social position and socio-economic status with the intervention, with child feeding seen as ‘women’s business’. Some caregivers reported that proper sanitation or water was not available to handle RUSF in a hygienic manner in some cases.

**Theme 3.3: Caregivers do not always have control over providing ready-to-use supplementary food for infant and young child in the intended amount because of sharing practices (direct and indirect evidence):** Caregivers indicated that they understand that sharing RUSF is counterproductive and that the whole ration is needed for the intended child. However, caregivers reported that some reasons out of their control resulted in RUSF being shared: these included gender (fathers and husbands instructing caregivers to give supplements to other children), seniority in the household (senior co-wives demanding RUSF for themselves or their children), social capital (sharing RUSF with neighbours with the understanding that this would be reciprocated in a time of need), as well as older children taking rations when caregivers are not at home.

**Theme 4.1: Fortified blended foods are thought to be good for children and are considered medicines by some, but sensory attributes and burden of preparation may affect acceptability (direct and indirect evidence):** Caregivers felt that the nutritional supplements improved the appetite, vitality, health, activity, physical and cognitive development of their children. Some caregivers reported that grandmothers suggested to them that they should have their child enrolled in the programme providing FBF because of concern that the child’s weight is low. Caregivers also experienced FBF as relieving the financial burden on the household and appreciated the reduction in medical costs because of their children’s improved health. Some caregivers believed that the FBFs are medicine and Western food. Perspectives suggest that these caregivers believe that FBFs are local foods that have been ‘transformed’ into foods of higher effectiveness. Because of its perceived effectiveness and the perception of FBF being medicine, most caregivers did not share the FBF with other members of the household, although some did indicate that they gave small quantities to other children. In addition, breastfeeding mothers also consumed some of the FBF intended for their children as they believed it increased their milk production. While caregivers found preparation easy, they experienced it as a burden on their time in cases where their children rejected the FBF. Caregivers reported that children enjoy the taste of FBF; some children disliked it initially, but they grew to accept it. However, most children did not like the smell of the CSB as it reportedly smells strongly like medicine. Caregivers reported that not all children enjoy the taste, with caregivers reporting that the FBF is too sweet for their children. Furthermore, caregivers reported that the colour and texture of the porridge changed after some time and found this unappealing. Many caregivers reported that they served FBF raw or ‘moistened’, as they would when preparing local couscous, based on the child’s preference and sometimes contrary to the instructions for preparation.

**Theme 4.2: Education may facilitate fortified blended foods use, while social factors, taste and quality, as well as limited sanitation, may be barriers to implementation (direct and indirect evidence):** The need for information and education sessions as facilitators to implementation was highlighted, with researchers noting the impact of these on the consumption of the supplement. Some barriers to effective and safe implementation were also identified. Gender roles, social position and socio-economic status interacted with the intervention and affected implementation, with child feeding seen as ‘women’s business’. Caregivers reported that proper sanitation or water was not available to handle FBF in a hygienic manner in some cases. Some caregivers also reported that FBF did not taste good as it was bitter and old; this was attributed to the flour being old and spoilt when they received it. Caregivers themselves reported that they did not always have the proper sealed containers to keep FBF fresh and pest-free.

**Theme 4.3: Caregivers do not always have control over providing fortified blended food to intended infant and young child only (direct and indirect evidence):** Caregivers indicated that they understand that sharing FBF is counterproductive and that the whole ration is needed for the intended child. However, caregivers reported that some reasons out of their control resulted in FBF being shared: these included culture within the household (co-wives demanding FBF for themselves or their children) and social capital (sharing FBF with neighbours with the understanding that this would be reciprocated in a time of need), as well as older children taking rations when caregivers are not at home.

**Theme 5.1: If adequately informed, caregivers mostly consider *child micronutrient supplementation* to benefit child well-being and be convenient (direct and indirect evidence):** Caregivers experienced that the MNS improved the health, appetite, energy as well as physical and cognitive development of their children. In addition, it was perceived by caregivers that MNS helps to prevent anaemia in children, thereby reducing the need for ‘expensive foods’. Although some caregivers did report negative gastrointestinal side effects, this was considered to be a sign that the intervention is working. These experiences were key to the acceptability of MNS as education alone would not have resulted in such a high level of acceptability. Caregivers found that the appearance of MNS makes it easy to give to children, as the white colour made children think it is sugar; the colour also makes it easy to blend into rice. However, some caregivers reported that they were not sure how to mix the MNS with food, and that they did not know what the MNS was, while some caregivers indicated distrust of the intervention and its rationale.

**Theme 5.2: Misconceptions of intervention providers and poor communication hinders implementation of *child micronutrient supplementation* (direct and indirect evidence):** Most intervention providers felt that MNS distribution increased their workload. However, the acceptable taste and ease of administration of MNS, as well as the benefits to children, made this an acceptable intervention from the perspective of most intervention providers, and as a result most did not mind the additional burden. However, some intervention providers from a study reporting indirect evidence did not seem to understand the intervention themselves: misconceptions around the types of foods that MNS should be mixed into as well as a lack of willingness to use educational materials may be barriers to implementation from the intervention providers’ side. Caregivers’ responses about the types of foods that MNS can be mixed into echo the misconceptions of intervention providers. Caregivers expressed a need for more frequent MNS distribution as this would mean more contact with intervention providers ‘of whom they would be able to ask questions if they had any problems’. In the study where intervention providers indicated low levels of intervention coherence, caregivers indicated higher levels of distrust in MNS. This uncertainty around preparation and rationale, as well as reported gastrointestinal side effects of the product, may be barriers to implementation from the caregivers’ side.

**Theme 6.1: Perspectives on the tolerability and health benefits of *alternative food for mothers* were mixed (direct evidence):** Pregnant women had mixed feelings about the sensory attributes of the alternative food. There was general acceptance for the texture and taste, but the smell was less acceptable. There were mixed feelings about the health benefits of the alternative food for mothers and babies. Women indicated that they would use the alternative food daily during pregnancy as the main belief was that it was good for the baby only.

**Theme 7.1: Acceptability of and adherence to small-quantity lipid-based nutrient supplement *for mothers* was affected by sensory experiences, perceptions on side effects and beliefs about health benefit (direct evidence):** Pregnant and lactating women (PLW) had mixed feelings about SQ-LNS, with some experiencing the taste and smell as unpleasant, while others reported that it tastes good. Some women also perceived that SQ-LNS causes side effects during pregnancy, including vomiting. These experiences and perceptions affected adherence. Intervention providers agreed with the perspectives of PLW around the challenging taste and smell of SQ-LNS; this perceived acceptability may have impacted on feasibility. Even though some women did not enjoy the SQ-LNS, they reported that they continued using it, as they believed the nutrients in it to be beneficial to their own health and strength as well as the health and intelligence of their unborn babies. However, some mothers reported that, after the birth of their child, they consumed less SQ-LNS, or did not have time to eat it, as they perceived that it does not provide the same benefits to their child post-partum.

**Theme 7.2: *Mothers* do not always trust unfamiliar or foreign small-quantity lipid-based nutrient supplement food products, which impacts adherence (direct and indirect evidence):** Foreign SQ-LNS, provided in packaging with a language that was not understood by PLW, was treated with scepticism and curiosity. This resulted in some women destroying the product, while some women shared the product with other pregnant or lactating women. Intervention providers reported that some PLW were not home to receive the product, some did not take it because of monotony or ‘laziness’ to do so, and some avoided it because of adverse effects related to the smell, such as vomiting.

**Theme 8.1: Acceptability of ready-to-use supplementary food *for mothers* was affected by sensory experiences and perceptions on side effects although beliefs about health benefits improved adherence (indirect evidence):** Pregnant and lactating women accepted the taste and texture of the RUSF with docosahexaenoic acid (DHA), although some reported not enjoying the taste. Most women reported a strong dislike for the fishy smell. Even though some women did not enjoy the RUSF, they reported that they continued using it, as they believed the nutrients in it to be beneficial to the health and intelligence of their babies. As pregnant women were considered to be more sensitive to certain foods and smells, the RUSF was perceived to be more tolerable after birth.

**Theme 9.1: Uptake of *maternal micronutrient supplementation* is poor because of its taste, side effects, competing priorities and misconceptions around the intervention (indirect evidence):** Some PLW experienced iron–folic acid supplements as having a bad taste and causing side effects such as nausea and vomiting. As a result, some did not like to take the supplement, while others stopped taking it entirely. Some perspectives suggested taking the supplements might also be a burden: participants were evenly divided on whether taking the tablet or pill with a meal is difficult; some reported forgetting to take these supplements because of household chores, such as childcare. Those who considered taking the supplement to be easy had mostly already adopted the behaviour. Some perspectives suggested that uptake is affected by a lack of understanding of the intervention. Perspectives of women suggested the discontinuation of supplementation was because of lactating or concerns that the foetus would grow too large and complicating delivery: ‘My mother told me the baby will grow too big and that taking too many tablets is not good for pregnant women’.^60^

**Theme 9.2: A lack of prioritisation of maternal malnutrition affects feasibility of providing *maternal micronutrient supplementation* (indirect evidence):** A lack of understanding and low awareness of the severity and consequences of maternal malnutrition at both the HCW and beneficiary level were identified by intervention providers as serious barriers. These shortcomings resulted in low prioritisation and poor implementation from the intervention provider’s side, as well as poor uptake and a lack of interest from the side of PLW.

Intervention providers further highlighted weak systems in terms of programme management, procurement, logistics and information management, as well as constrained resources such as buildings, staff and equipment, as barriers to reaching vulnerable populations to improve maternal malnutrition.

**Theme 9.3: Differential access to *maternal micronutrient supplementation* caused inequity (indirect evidence):** Pregnant and lactating women reported that travelling far from remote areas to healthcare clinics is challenging, with some discontinuing supplementation for this reason. Some also reported that financial constraints, caste and tribal discrimination and the low social standing of women resulted in differential access to services.

**Theme 10.1: Community involvement in the production of *alternative foods for mothers and children* can ensure sustainability (indirect evidence):** Intervention providers expressed that buy-in from communities is essential to ensure the sustainability of a programme providing pregnant women and young children with alternative foods. Encouraging the production of local foods for this purpose was identified by intervention providers as an approach, although some experiences indicated that this has to be done sensitively as certain plants may have negative connotations for local communities. For example, health promoters encouraged the cultivation of a local spinach-like plant without realising that it ‘… is considered by the local people to be an aphrodisiac, which leads to a reluctance to openly farm this plant’.^52^

**Theme 10.2: Intra-household food distribution is a cultural practice that affects intervention adherence to *alternative food for mothers and children* (indirect evidence):** Caregivers and pregnant women relayed experiences around the sharing of food among family members. Some noted specifically that cultural issues determine who gets food first, while others reported that poverty often necessitated that wage-earning family members eat first to ensure they can work.

**Theme 11.1: *PLW* may not always tolerate small-quantity lipid-based nutrient supplement, but most *children* tend to enjoy it (direct evidence):** Some PLW experienced the texture, taste and smell of SQ-LNS as unpleasant. Women frequently reported vomiting and nausea resulting from the smell and taste of the SQ-LNS. This was particularly true for pregnant women, and more so for women during early pregnancy, as it was reported that these experiences were diminished post-partum or later in the pregnancy. Some women even reported eating SQ-LNS directly from the sachet as a snack later in the study. Researchers concluded ‘the majority of the nausea and vomiting experienced by the women was because of their physiological state in early pregnancy’.^46^ However, a small proportion of women continued to experience nausea and vomiting even post-partum. Specific comments relating to sensory dislikes of SQ-LNS suggested that the taste and smell of groundnut were not acceptable to some, while others felt it was too sweet or bitter. Some women reported that they did not mind the sensory attributes of SQ-LNS as long as it is beneficial to their health. Ways of overcoming the negative sensory experiences included mixing SQ-LNS into porridge and adding sugar to it. Despite these, some women would not use SQ-LNS when they did not have the food they liked mixing it into, and most women described incidents of non-adherence. In contrast, caregivers of children reported that they liked the colour and consistency of SQ-LNS. They also experienced that their children did not refuse to eat SQ-LNS and liked the taste and smell of it. However, some caregivers reported that children would enjoy SQ-LNS more if it were sweeter, and one woman suspected that her child did not like the supplement because of the smell. One woman reported that her child had vomiting and diarrhoea after eating SQ-LNS. Most caregivers approved of the consistency of SQ-LNS as it was easy for children to eat and easy to mix into hot rice, although a minority had trouble mixing it with rice.

**Theme 11.2: Experiences of self and others influence the uptake of small-quantity lipid-based nutrient supplement *for mothers and children* (direct evidence):** Unrealistic expectations of cause and (immediate) effect, likely because of past experiences, as well as attribution of events, rightly or wrongly, to SQ-LNS also influenced uptake. On the one hand, women expressed concern that taking nutritional supplementation could lead to large babies and obstetrical complications. This was based on beliefs of ‘women in the community’, suggesting that this perception may be anecdotal.^53^ On the other hand, some women did not see the need for supplementation as ‘children grew normally, whether the mother took supplements or not’,^46^ indicating an expectation that visible growth should be seen nearly immediately.

**Theme 11.3: People interact differently with small-quantity lipid-based nutrient supplement *for mothers and children* based on whether they believe it to be medicine or not (direct evidence):** A theme emerging strongly from the quotes indicated that many beneficiaries believed that SQ-LNS is medicine. Researchers reported that in Ghana, women typically referred to Nkatepa as ‘blood tonic mixed with groundnut paste’, and in Malawi, women said ‘… there is medicine in Chiponde, to increase blood in the body’.^46^ This perception was reinforced by instructions on SQ-LNS use being given by nurses or doctors at health facilities; many women had memorised the instructions for SQ-LNS correctly and reported that they consumed SQ-LNS according to these instructions. However, researchers reported that several caregivers opined that SQ-LNS should be promoted as ‘special food’ and not ‘medicine’. These contradictory positions seemed to drive the way in which beneficiaries interacted with SQ-LNS. Several PLW had negative sensory experiences of SQ-LNS as they said they could smell or taste the ‘medicine’ in the product or feel the particles of ‘blood tonic’ on their tongue. Some who could not tolerate its organoleptic properties reported going to buy Pregnant Care vitamin and mineral supplement from the hospital. This practice may have been based on another reported perception that SQ-LNS should replace all usual pregnancy medications. Other women compared their experiences of SQ-LNS with earlier pregnancies when they took medicine (such as vitamin B complex); some considered SQ-LNS better, while others preferred pills and tablets. Pregnant and lactating women reported not using SQ-LNS when they had been to the hospital for an injection, stating that they do not take SQ-LNS together with injections. Pregnant and lactating women experienced health improvements and increased energy and felt that they had more breast milk to feed their children. Most caregivers perceived that the SQ-LNS was good for their children through improvements in the health, energy and appetite of the child.

**Theme 11.4: Gender roles and cultural sharing practices may result in small-quantity lipid-based nutrient supplement *for mothers and children* not reaching its intended beneficiaries (direct evidence):** Cultural sharing practices were commonplace and reported as being shared with both adult sisters of PLW as well as their children in the matrilineal Malawian society.

Women did not have autonomy in decision-making about taking SQ-LNS or giving it to their children but had to ask their husbands. Beliefs about whether SQ-LNS was medicine influenced whether husbands allowed women to take it. Beneficiary women expressed that all women should receive SQ-LNS, and that poor women should be prioritised – this was in the context of a trial giving SQ-LNS to a random sample of eligible participants.

**Theme 12.1: Information about *multiple micronutrient powders (MMPs) for mothers and children* and its purpose is important for acceptance (indirect evidence):** Beneficiaries in the refugee camp expressed a need to understand what micronutrient powder (MMP) is and what it is used for before they would use it. The confusion around its purpose was seen in perspectives attributing reduced aches and pains, easing of childbirth, trouble with sleeping and being hungry to the product. Distrust of the product came through strongly because of a lack of understanding and because it was not delivered by health providers but rather the World Food Programme (WFP). It should be observed that these perspectives come exclusively from a displaced group of respondents. Healthcare providers agreed with beneficiaries that distribution of MMP by the WFP, instead of by healthcare providers, is inappropriate and unacceptable. Insufficient information about MMP disempowered them from dispelling beneficiary distrust, and therefore some gave discouraging information to beneficiaries.

**Theme 12.2: A lack of information and cultural/religious sensitivity may exacerbate inequity in the provision of *maternal and child micronutrient supplementation* (indirect evidence):** A lack of information on the ingredients of MMP led people of the Muslim faith to be concerned about the constituent elements and whether these were halal. A participant from Somalia reported that some residents of the refugee camp believed that the MMP was made of meat, adding that haram foods would be discarded. In addition, the packaging depicted a genie- or ghost-like figure that was seen as offensive to people of the Islamic faith and discouraged its use among Muslim people in the camp.

### Perspectives about coverage of the interventions

A single article reported on both blanket and targeted intervention delivery in the same country; however, it did not report any comparative perspectives related to coverage.^38^ A few other articles included in different intervention categories reported some perspectives around coverage, even though targeted and blanket approaches were not both studied in these articles. These were very sparsely reported and synthesis could not be conducted; therefore, findings should be cautiously interpreted.

#### Infant and child supplementation

For child MNS, direct evidence from an article providing MMP to infants and young children in urban and rural Bangladesh provided perspectives on acceptability of this approach.^53^

#### Maternal and child supplementation

**Maternal and child food supplementation:** Direct evidence from an article providing SQ-LNS to infants and young children as well as PLW in urban and rural Bangladesh provided perspectives on acceptability and feasibility of this approach.^53^ While the supplement was provided through blanket distribution with no nutritional eligibility criteria, it is important to note that participants were randomly selected to participate in the study.

**Maternal and child micronutrient supplementation:** Indirect evidence from an article providing MMP to all residents of a refugee camp in North-western Kenya provided perspectives on acceptability of a blanket delivery approach.^47^ It is important to note that residents of the camp as well as the local Turkana population in this semi-arid region also received food rations as part of a separate support package, although the MMP was distributed by making use of the first cycle of the twice-monthly ration distribution.

## Discussion

This article describes the identification and synthesis of the perspectives of key stakeholders regarding the acceptability, feasibility and equity of nutritional supplementation interventions for the prevention of wasting in infants and young children, as well as perspectives on the delivery approach of these interventions. This work is embedded within a larger QES for WHO guideline recommendations on the prevention of wasting in IYC under 5 years of age.

### Infant and child supplementation

Caregivers’ acceptance of IYC supplementation was affected by several factors. Our findings that some interventions are enjoyed by IYC following initial dislike confirm those of Schlossman et al., who found high taste acceptability of RUSF regardless of dairy protein content in Guinea-Bissau and Phuka et al., who reported on acceptability of SQ-LNS in Malawi.^79,80^ However, taste is likely to be highly influenced by the constituent ingredients, with another study conducted in Cambodia reporting that while a locally produced RUSF made with small freshwater fish instead of milk powder was deemed acceptable, several caregivers remarked on its fishy smell.^81^ Furthermore, formative research conducted in Mexico indicated that unsweetened SQ-LNS is not largely accepted.^82^ Perspectives around sensory attributes affecting the acceptability of FBF confirm the findings of a study in Tanzania, which also demonstrated that sensory characteristics of novel FBFs, as well as CSB+, can affect acceptability, although liking may increase over time.^83^ We found several facilitators of providing nutritional supplementation; however, most of these can only improve feasibility if resources are available, and nutrition care for malnutrition has been shown to be constrained in low-resource settings.^84^ In addition, some barriers, such as social factors and poor sanitation, cannot be overcome by these facilitators alone and will need to be considered during programme implementation. Sparse evidence regarding equity also indicated factors that would also need to be considered during programme implementation.

### Maternal supplementation

With regard to acceptability, perspectives indicated that maternal nutritional supplementation had mixed acceptability in terms of sensory attributes. This confirms formative work done with pregnant women in Burkina Faso that indicated sweet energy–protein supplements were preferred to savoury products and that several participants reported sensitivity to smell and taste that made them nauseous.^85^ It should be observed that acceptability may be very context-specific, as pregnant women in Ghana reported double the willingness to pay for SQ-LNS when compared to those in Malawi.^86^ Sparse perspectives on feasibility indicated that implementation is impacted by a lack of prioritisation of maternal malnutrition, likely exacerbated by a distrust of unfamiliar food or products. These findings are similar to those among nutritionally vulnerable lactating mothers in Ethiopia, where maternal malnutrition was found to be associated with social and psychological factors as well as dietary knowledge.^87^ Very sparse evidence regarding equity indicated that differential access to MNS need to be considered during programme implementation.

### Maternal and child supplementation

Perspectives around the acceptability and equity impact of interventions in this category were similar to those for interventions targeting these groups separately. However, it additionally emerged that the acceptability, and consequent uptake, may be influenced by experiential understanding of the interventions as well as whether caregivers believed the interventions were medicine or not; inequity might also be exacerbated by gender roles, religious sensitivity and cultural sharing practices. Encouragingly, a recent randomised controlled trial in Pakistan showed limited impact of sharing practices on the linear growth and nutritional status of children.^88^ There was very little information regarding feasibility, but perspectives around alternative foods suggested that community involvement in the food production may act as facilitators.

### Strengths and limitations of the review

We conducted a rapid, fit-for-purpose QES on nutritional supplementation to inform WHO guideline recommendations on the prevention of wasting in children. It was positively received as meeting the requirements of the GDG and has been included in the WHO guideline, which is published and accessible at https://app.magicapp.org/#/guideline/noPQkE/section/jzoeJg (Section 4D of the document addresses the prevention of wasting in IYC). To ensure that the results could be directly used in the EtD framework, we presented findings per intervention category and phenomena of interest. This led to a loss of richness of pertinent cross-cutting themes as well as sparse data in some intervention categories. We drew informally on a published rapid Cochrane QES, but did not follow formal methodological guidance such as recently published guidance on the conduct of rapid QES.^89^ This guidance was not available at the time of conducting the QES. Although we used the best-fit framework synthesis approach to synthesise data, there were some amendments to the proposed approach.^10,11^ Because of time limitations and the rapid nature of the review, we used a snowball approach, rather than a systematic search, to identify relevant frameworks. Furthermore, as all data fit into the *a priori* coding framework, we did not code inductively. Our themes therefore all emerged from the framework synthesis stage and not from thematic synthesis, and we did not create a new model based on our findings. Our coding and synthesis can be seen as another layer of interpretation of the perspectives of participants and findings of authors of the primary qualitative studies. However, this is a step that is inherent to QES, and any bias was minimised through team consensus meetings. Furthermore, trade-offs in terms of rigour were required to achieve this within the policy window. Firstly, our search was restricted to one database to ensure a manageable yield. While we acknowledge that searching one database is not exhaustive, MEDLINE is one of the largest searchable biomedical databases. Furthermore, we applied no restrictions to our search and supplemented the search yield with hand searches for qualitative ‘sibling’ studies of trials of effectiveness as well as through contacting experts; therefore, we consider the search to be comprehensive. Secondly, we conducted a ‘pre-screening’ step to ensure studies were describing the correct indication and intervention prior to extensive full-text screening. Thirdly, we restricted eligible interventions to those included in the systematic review of effectiveness. Fourthly, we limited perspectives to those of two stakeholder groups and restricted perspectives of HCWs to those around feasibility. Finally, we did not assess the certainty of the evidence as this is not explicitly considered under the EtD domains. Our search for the QES was conducted on 13 June 2022, and we acknowledge that perspectives around nutritional supplementation for the prevention of wasting may have shifted. However, after conducting an updated search and rapid appraisal of recently published studies (until 23 December 2024), we identified seven studies that supported our findings, with no new findings emerging.^90,91,92,93,94,95,96^

## Conclusion

We were able to conduct a rapid QES on the acceptability, feasibility and equity considerations of nutritional supplementation for the prevention of wasting in children. Our findings indicated that nutritional supplementation interventions were probably acceptable. We identified several facilitators of intervention implementation; however, some barriers that would need to be considered during programmatic implementation were also identified. Information regarding equity was relatively sparse, and the impact of the provision of nutritional supplementation remains unclear.
